# Special Issue—“Orthopedics and Coronavirus: Analyze the Past to Face the Present and to Prevent the Future”

**DOI:** 10.3390/jcm11216440

**Published:** 2022-10-30

**Authors:** Giuseppe Maccagnano, Francesco Maruccia, Nicholas Elena, Vito Pesce

**Affiliations:** 1 Orthopaedics Unit, Department of Clinical and Experimental Medicine, Faculty of Medicine and Surgery, University of Foggia, Policlinico Riuniti di Foggia, 71122 Foggia, Italy; 2 Fondazione Casa Sollievo Della Sofferenza IRCCS, 71013 San Giovanni Rotondo, Italy; 3 Department of Orthopedics and Trauma Surgery, University of Verona, 37129 Verona, Italy

The coronavirus pandemic represented one of the most massive health emergencies in recent history [1]. COVID-19 started in Wuhan, China, rapidly spreading worldwide, causing millions of deaths and deep social–economic wounds [2]. The COVID-19 disease was caused by a coronavirus, named SARS-CoV-2. The affected patients mainly presented a self-limiting flu-like syndrome. At the same time, the elderly and fragile part of the population could develop acute respiratory distress syndrome (ARDS), multi-organ failure (MOF), and sometimes even lead to exitus. This condition necessitated a higher need for hospitalization in infected patients, especially in intensive care units (ICUs) [3]. The SARS-CoV-2 pandemic caused devastating changes in the dynamics of national health systems; both public and private hospitals were reorganized to treat the vast number of patients with the COVID-19 disease, thus leading to a total stop of nonurgent elective surgeries, including orthopedic procedures [4]. Many healthcare professionals, including orthopedists, underwent numerous training activities during the pandemic, often very distant from their regular field of work, and then were re-employed to manage patients with COVID-19. As already stated, this resulted in a substantial reduction in elective orthopedic surgical activity. Nevertheless, the number of fractures worthy of surgery has remained constant. Their treatment has been more complicated due to the difficulties associated with the new operating room setting and patients’ health conditions.

Orthopedic surgery generates droplets and aerosols, and it is a concern amongst surgeons that otherwise rational precautionary principles are being set aside due to a lack of scientific evidence as well as a shortage of personal protective equipment (PPE) [5].

To better realize this field’s evolution, this Special Issue in the *Journal of Clinical Medicine* aims to investigate and cover recent as well as novel advancements, in addition to future trends, during a pandemic in the orthopedic field.

A lot of exciting news has been published in this Special Issue. Following recent literature, Covino et al. [6] demonstrated how during the first year of the COVID-19 pandemic, despite a lower rate of injuries to the hand, there was a higher percentage of surgical intervention on that specific body part. The lockdown favored housework and home improvements, leading to different and more severe traumas, especially in the elderly. Therefore, the authors suggested heavy investments in telemedicine, foreseeing possible future pandemics. Specifically, Hsu et al. [7] investigated the effects of telemedicine during this pandemic. They stated that orthopedic surgeons must implement it in a better, more flexible, and accessible way. The need for preventive strategies and algorithms for surgical planning was evident. Moreover, even in nations where a lockdown was not implemented, the number of patients willing to undergo surgery, especially elective procedures, greatly declined. Taiwan is an example of a nation that had less restrictive safety measures. Hsu and Hsu [8] blamed the decrease in surgical procedures on patients’ fears due to this worldwide condition. Mental health must not be overlooked in the future, especially in cases of future pandemics, in order to lead our patients toward better treatment choices. A recent study by Mastri et al. [9] observed a decrease in day-one post-operative fever in patients wearing PPE. In addition, the prevention of airborne diseases allows healthcare professionals to recognize post-operative complications causing fever, such as infections. It also significantly affects the costs and hospital stays of our patients. Last but not least, this workgroup analyzed the impact of different surgical hip approaches in COVID-19 patients [10]. The lateral approach by Hardinge safeguards the respiratory function in patients with moderate and severe gas exchange rate impairment compared to the direct anterior approach (DAA). Meanwhile, it does not affect patients’ respiratory function in mild impairment cases. The ASA score is also a valuable tool in predicting post-operative risk. Patients with four or more comorbidities present a higher risk of exitus in the post-operative period. After so many contributions, it is evident how the pandemic sped up the transition to telemedicine and, at the same time, highlighted the importance of mental healthcare for the population. Despite this, many structural, political, and surgical matters still need to be investigated to prepare for future pandemics.

As the Guest Editor, I would like to give special thanks to the members of the *JCM* Editorial Office for their essential support and to the reviewers for their professional and appropriate comments. Finally, I would like to praise the authors’ efforts and contributions. I genuinely believe that this Special Issue will be stimulating and will inspire confrontation in the present to be better prepared for the future.
